# Closed-loop transcutaneous auricular vagus nerve stimulation for the improvement of upper extremity motor function in stroke patients: a study protocol

**DOI:** 10.3389/fneur.2024.1379451

**Published:** 2024-06-05

**Authors:** Xue-Zhen Xiao, Rongdong Li, Chengwei Xu, Siqi Liang, Meng Yang, Haili Zhong, Xiyan Huang, Jingjing Ma, Qiuyou Xie

**Affiliations:** ^1^Zhuhai Fudan Innovation Institute, Zhuhai, Guangdong, China; ^2^BrainClos Co., Ltd., Shenzhen, Guangdong, China; ^3^Department of Rehabilitation Medicine, Zhujiang Hospital of Southern Medical University, Guangzhou, Guangdong, China; ^4^Rehabilitation Medicine School of Southern Medical University, Guangzhou, Guangdong, China; ^5^School of Biomedical Engineering, Shenzhen University, Shenzhen, Guangdong, China

**Keywords:** stroke, transcutaneous auricular vagus nerve stimulation, VNS, motor rehabilitation, electromyography, closed-loop, heart rate variance

## Abstract

**Background:**

Transcutaneous auricular vagus nerve stimulation (taVNS) has garnered attention for stroke rehabilitation, with studies demonstrating its benefits when combined with motor rehabilitative training or delivered before motor training. The necessity of concurrently applying taVNS with motor training for post-stroke motor rehabilitation remains unclear. We aimed to investigate the necessity and advantages of applying the taVNS concurrently with motor training by an electromyography (EMG)-triggered closed-loop system for post-stroke rehabilitation.

**Methods:**

We propose a double-blinded, randomized clinical trial involving 150 stroke patients assigned to one of three groups: concurrent taVNS, sequential taVNS, or sham control condition. In the concurrent group, taVNS bursts will synchronize with upper extremity motor movements with EMG-triggered closed-loop system during the rehabilitative training, while in the sequential group, a taVNS session will precede the motor rehabilitative training. TaVNS intensity will be set below the pain threshold for both concurrent and sequential conditions and at zero for the control condition. The primary outcome measure is the Fugl-Meyer Assessment of Upper Extremity (FMA-UE). Secondary measures include standard upper limb function assessments, as well as EMG and electrocardiogram (ECG) features.

**Ethics and dissemination:**

Ethical approval has been granted by the Medical Ethics Committee, affiliated with Zhujiang Hospital of Southern Medical University for Clinical Studies (2023-QX-012-01). This study has been registered on ClinicalTrials (NCT05943431). Signed informed consent will be obtained from all included participants. The findings will be published in peer-reviewed journals and presented at relevant stakeholder conferences and meetings.

**Discussion:**

This study represents a pioneering effort in directly comparing the impact of concurrent taVNS with motor training to that of sequential taVNS with motor training on stroke rehabilitation. Secondly, the incorporation of an EMG-triggered closed-loop taVNS system has enabled the automation and individualization of both taVNS and diverse motor training tasks—a novel approach not explored in previous research. This technological advancement holds promise for delivering more precise and tailored training interventions for stroke patients. However, it is essential to acknowledge a limitation of this study, as it does not delve into examining the neural mechanisms underlying taVNS in the context of post-stroke rehabilitation.

## Introduction

The vagus nerve, also referred to as cranial nerve X, stands as the most extensive cranial nerve within the human body. It plays a pivotal role in regulating a multitude of involuntary bodily functions, encompassing heart rate, respiration, digestion, and overall internal homeostasis, functioning as a crucial component of the parasympathetic nervous system (1, 2). Vagus nerve stimulation (VNS) is a method that involves stimulating the vagus nerve using an implantable device. In this procedure, an electrode will be available to the vagus nerve by some operation, and a small device is implanted in the chest to produce specific stimulation. VNS has gained the Food and Drug Administration (FDA) approval as an effective therapy for epilepsy in 1997, as well as for depression in 2005. The most recent FDA approval for VNS is in the realm of motor rehabilitation of upper extremities following ischemic stroke. In recent years, both invasive and non-invasive VNS have garnered increasing attention in the context of ischemic stroke, emerging as a promising new treatment (3).

Recent studies on rat models of stroke (4–9) and stroke patients (10–12) have demonstrated positive effects of VNS on motor rehabilitation. The study by Dawson et al. (10–12) using a randomized double-blind approach showed that the efficacy of VNS paired with motor training was two to three times than that of sham VNS combined with motor training on the upper extremity rehabilitation in ischemic stroke patients. Several mechanisms have been proposed to elucidate the rehabilitative effects of VNS on stroke. These mechanisms include the reduction of neuronal apoptosis (13), the mitigation of infarct size (14), the regulation of neurotransmitter release (15, 16), the modulation of pathways associated with inflammatory factors (17), the enhancement of neurocircuit plasticity (9), the change in the blood–brain barrier permeability (18), and the effects on the hemodynamics (19). In terms of specific mechanisms underlying movement-paired VNS, particularly within the context of the closed-loop VNS as defined in the current study, previous research has shed light on the enhancement and plasticity mechanisms. Dawson et al. (12) have suggested the augmentation of neurocircuit plasticity through VNS treatment when combined with motor rehabilitation training. In a study involving animal models, Meyers et al. (9) found that VNS coupled with rehabilitative training heightened the plasticity within corticospinal motor networks in rats with ischemic lesions, thereby amplifying synaptic connectivity to the musculature of the rehabilitated forelimb. Furthermore, Bowles et al. (15) demonstrated that the application of VNS immediately after a successful movement enhances motor learning through a cholinergic reinforcement mechanism, leading to the selective modulation of M1 neurons. These findings underscore that the impact and mechanisms of VNS on motor rehabilitation may depend on the strategic combination of targeted events, such as motor movements.

However, it should be noted that this invasive VNS requires expensive surgical procedures and has several contraindications (20). Consequently, researchers and clinicians are exploring a non-invasive VNS as a potential alternative intervention. Recently, the use of transcutaneous auricular vagus nerve stimulation (taVNS) as a non-invasive brain stimulation technique in ischemic stroke has received increasing attention [see the review (21)]. This innovative technique involves non-invasive electrical stimulation of the auricular branch of the vagus nerve (ABVN). It has been revealed that this branch projects upstream of the nucleus of the solitary tract (NTS) by traversing the vagal trunk and passing through the jugular ganglion (20, 21). The NTS projects directly or indirectly to a wide range of nuclei, from lower to higher regions, encompassing the parabrachial nucleus, dorsal raphe nucleus, locus ceruleus, hypothalamus, thalamus, amygdala, and hippocampus (1, 22–24). Subsequently, these projections extend further into the cerebral cortex. Compared to VNS, taVNS offers a low-risk, user-friendly, and economic intervention that eliminates the need for surgery and the associated postoperative complications (25, 26).

A small number of research have provided evidence to support the enhancement of motor rehabilitation in stroke patients after taVNS treatment (27–31). A recent meta-analysis has indicated that the efficacy of taVNS in upper extremity rehabilitation for stroke patients can be comparable to that of VNS (26). However, the number of studies is small, and specific taVNS treatment protocols vary. In the study of Wu et al. (31), stroke patients were randomly assigned to receive either real taVNS or sham taVNS, followed by transitional movement training. The taVNS group showed significant improvement in upper limb function, with a 6.9-point improvement in Fugl-Meyer Assessment of Upper Extremity (FMA-UE) and a 6.5-point improvement in Wolf motor function test (WMFT) after the treatment, compared to the sham taVNS group (3.18 and 2.91 points, respectively). In contrast, in another study by Chang et al. (28), each burst sequence of taVNS was administered concurrently with an upper limb movement by mechanical control, leading to significant improvement in spasticity, but no significant difference in FMA-UE (3.10 vs. 2.86) with sham group. Moreover, in another study by Bradan et al. (27), taVNS was administered concurrently with movement training in two different manners. In the paired condition, each burst sequence of taVNS was synchronized with an upper limb movement using an electromyography (EMG)-triggered taVNS system, i.e., a closed-loop system. In the unpaired condition, a programmed taVNS with chronological stimulation was initiated during the movement training but was not synchronized with movements. FMA-UE scores improved in both groups (5.00 vs. 3.14), with a slightly larger improvement observed in the paired group. Although no statistically significant differences were found from less than 10 patients in each group, this study highlights the potential of utilizing an EMG-triggered closed-loop taVNS system to achieve precise stimulation paired with each movement training. Although these findings indicate taVNS as a valuable tool for post-stroke rehabilitation of acute [0.5 month after stroke (28, 29)], subacute [3 months after stroke (28, 30)], and recovery [6 months after stroke (27, 29)] phases, it remains unknown whether the concurrent application of taVNS and motor movement training is critical for stroke rehabilitation. A comprehensive and random controlled clinical trial with a sufficient sample size is essential to directly validate the clinical effectiveness of taVNS when administered concurrently with motor movements through EMG-triggered closed-loop system. This could provide scientific and data support for the establishment and application of this novel hardware and software system, i.e., EMG-triggered closed-loop taVNS system, in stroke rehabilitation.

In this study, we propose to directly investigate the necessity of the taVNS applied concurrently with motor training for post-stroke rehabilitation. Additionally, we aim to validate the benefits of employing an EMG-triggered closed-loop system in the administration of taVNS treatment during motor training. To achieve this, we have designed two experimental conditions to investigate whether the efficacy of taVNS concurrently with motor movement, i.e., each taVNS burst sequence triggered by the EMG of each motor movement, is superior to that administered sequentially with motor movement. A sham control group has been incorporated to confirm the effectiveness of taVNS treatment. The significance of rehabilitation efficacy within the concurrent group, especially if it significantly surpasses that observed in the sequential and sham groups, would substantiate the necessity for applying taVNS concurrently with motor training as well as support the advantages of the EMG-triggered closed-loop system.

## Methods

### Participants

This study presents a protocol for a single-center randomized, double-blind controlled trial. To participate in this study, patients are required to meet following specific criteria: (1) Aging between 18 and 80; (2) Having a confirmed diagnosis of ischemic stroke by a qualified clinician in accordance with the guidelines in the Chinese Stroke Prevention and Control Guideline from 2021; (3) In the acute/recovery phase, defined as occurring 2 weeks after the onset of stroke, exhibiting stable vital signs, and showing no progression of the disease within 48 h during this period; (4) Having unilateral upper limb motor dysfunction, are identified as monoplegia or hemiplegia. The participation in this study should be subject to their voluntary cooperation and signing an informed consent form.

Participants will be excluded if they have impairments of upper limb function other than those caused by stroke (e.g., shoulder-hand syndrome), a documented history of psychiatrist-related diseases, severe impairment of cognitive function, inability to cooperate in the rehabilitation training, receiving other neuromodulation rehabilitation treatments simultaneously, presence of cranial metal implants, skull-based pacemakers, the presence of severe spasticity, other serious injuries to the upper extremities, cardiac arrhythmias or other cardiac abnormalities, a history of respiratory disease or disorder (including pneumonia, dyspnea, and asthma), uncontrolled epilepsy or history of epilepsy, a history of vasovagal syncope, or having other contraindications to taVNS.

This study has obtained approval by the Medical Ethics Committee, affiliated with Zhujiang Hospital of Southern Medical University for Clinical Studies (2023-QX-015) and has been registered on ClinicalTrials (NCT05943431).

### Sample size

Sample size was determined using n = 2*[(Z_α/2_ + Z_β_)*σ*/*d*]^2^ and statistical power analysis software G*Power 3.1. Referencing the size (effect size, Cohen’s *d* = 0.632) and parameters of *α* = 0.05, *β* = 0.85 observed in the experiment of Dawson et al. (12), the estimated sample size for each group in this study should be 40. Considering the clinical dropout rate of 20%, the sample size was adjusted to 50 for each group.

### Randomization and blinding method

A total of 150 participants will be recruited and randomly assigned to three groups (Group 1, Group 2, or Group 3) in a 1:1:1 ratio. Participants will be instructed to randomly select a sealed envelope which contains a digital number ranging from 1 to 150. Those who draw numbers between 1 and 50 will be assigned to Group 1, 51 and 100 will belong to Group 2, and 101 and 150 will be designated to Group 3. The number in the envelope will only be revealed after the follow-up assessments to ensure the blinding of the allocation. This process will be overseen by a research assistant who will not involve in the intervention or data analysis phases. To ensure double-blinding, we will implement three procedures. Initially, we will utilize the same software across all tests, with the number drawn by the patient corresponding to different software settings. These settings will reflect the three distinct conditions. Further, all patients across the three groups will be outfitted with identical EMG sensors and taVNS stimulators. Lastly, we will guide therapists to activate the taVNS three times via a remote control, prior to each type of motor movement training. Consequently, patients in all three groups, particularly the sham group, will experience the sensation of ear stimulation.

### Procedures

Each participant will receive 14 treatment sessions on a daily basis for 14 days. During each session, participants will engage in motor movement tasks based on their occupational training protocol prescribed by their therapist. The motor movement tasks are derived from a comprehensive training pool of Dawson et al.’s experiment (2020, 2021, 2023) and consisted of six types of movement tasks, including grasping training, forearm rotations, gross movement training, fine motor training, feeding training, pinching and griping training. Within each type, there are two to four sub-items tailored for specific functional training purposes. To facilitate and standardize the movement tasks, appropriate aids were designed, and instructional videos were filmed in which the therapist demonstrated each movement task with her left or right hand at the appropriate speed. The duration of each video is approximately 6–7 s.

In Group 1 (concurrent condition), taVNS will be precisely synchronized with motor movements in an EMG-triggered closed-loop system. In Group 2 (sequential condition), participants will receive a session of taVNS treatment followed by motor movement tasks, and the sham taVNS will be paired with motor movements. Lastly, for participants in Group 3 (control condition), sham taVNS will be synchronized with motor movements.

Prior to each session, the therapist will adjust the motor training tasks and determine the number of repetitions for each movement based on each participant’s individual abilities and physical condition. Typically, each session will consist of 3–4 tasks, each task comprising 2–3 sub-items. In total, participants are expected to complete approximately 240–300 movements throughout each session.

**Figure 1:**
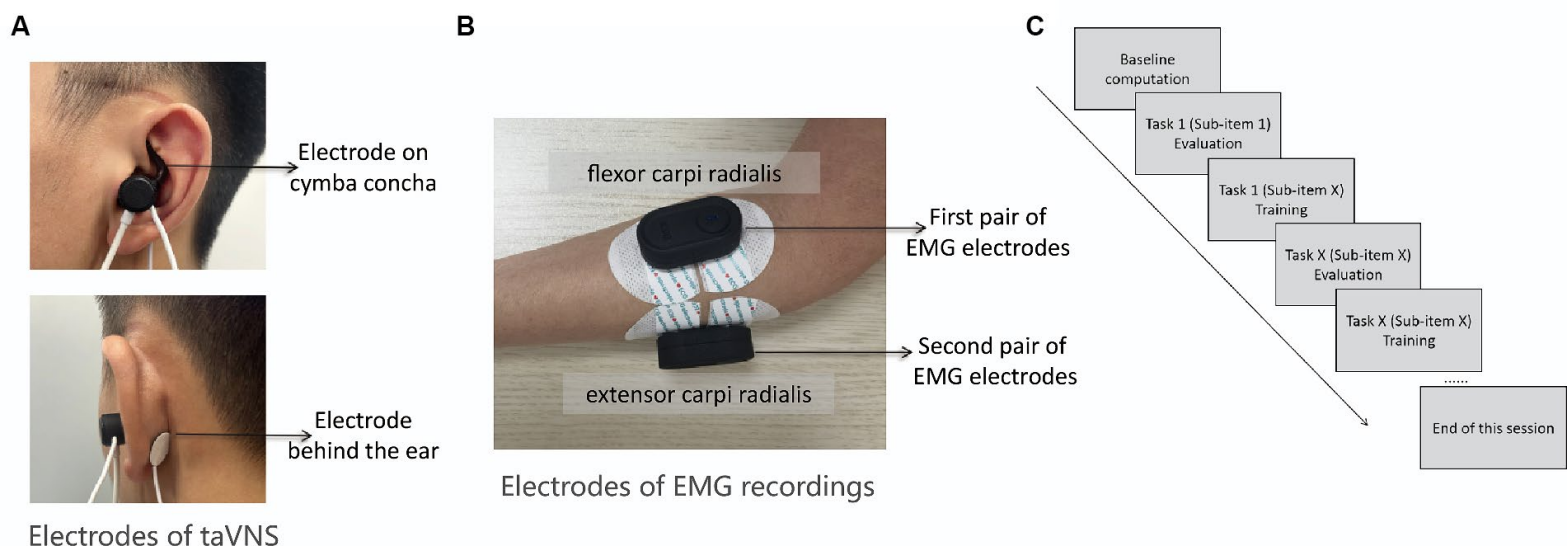
**(A)** The target area of taVNS is ABVN marked by the red ellipse. One of the stimulation electrodes (i.e., the tip of the earplug-shape device) will be fixed on the cymba concha and the other electrode will fixed on the position above the back of earlobe as shown by the arrows above and below; **(B)** Two pairs of wireless EMG electrodes will be attached to the extensor carpi radialis and flexor carpi radialis, respectively; **(C)** Diagram shows the procedures of each session.

The motor movement evaluation and training procedures are consistent across all three groups of patients. The experimenter will oversee the participant’s adherence to instructional videos when they replicate the movements using affected limb. Prior to each session, the participant will seat in front of a computer, positioning both upper limbs comfortably on the table. The experimenter will sterilize the cymba concha area of the participant’s left ear (as shown in Figure 1A) with an alcohol wipe. Subsequently, the taVNS stimulator will be affixed to the left ear, and the electrodes will be firmly attached on both the cymba concha area and the back of the ear. The cymba concha area is considered to be the primary distribution region of the ABVN (25, 32). The amplitude of the taVNS will be adjusted to remain below the patient’s pain threshold.

Subsequently, the experimenter will affix two pairs of wireless EMG electrodes to the extensor carpi radialis and flexor carpi radialis muscles of the affected arm to record surface EMG activity during the motor training sessions (as depicted in Figure 1B). The basic quality of EMG signal will be assessed when participants are at rest, and the signal’s fidelity during muscle contraction will be visually examined as participants grasp a ball with force. During rest, the EMG signal should exhibit minimal noise, whereas during muscle contraction, EMG spikes corresponding to muscle activity should be evident. Any deviations from these criteria will prompt adjustments to the position of EMG electrodes. With these preparations verified, the experimenter will commence the session by activating a button to calculate the baseline noise level of the EMG signal. The baseline noise level is derived from a 1.5-s period of EMG signals without any voluntary movement before evaluation. Subsequently, participants will be guided through the evaluation and training of each sub-item of the movement task using instructional videos in the software (as shown in Figure 1C). The evaluation before each movement training is to establish the individualized threshold for the subsequent training. The protocol reported by Badran et al. (27) using the EMG-triggered closed-loop taVNS system did not consider the individual EMG thresholds, whereas they input a constant threshold value for the initiation of taVNS. This could sabotage the efficacy of the taVNS due to the differences of threshold parameter from different participants and different motor movements.

**Figure 2:**
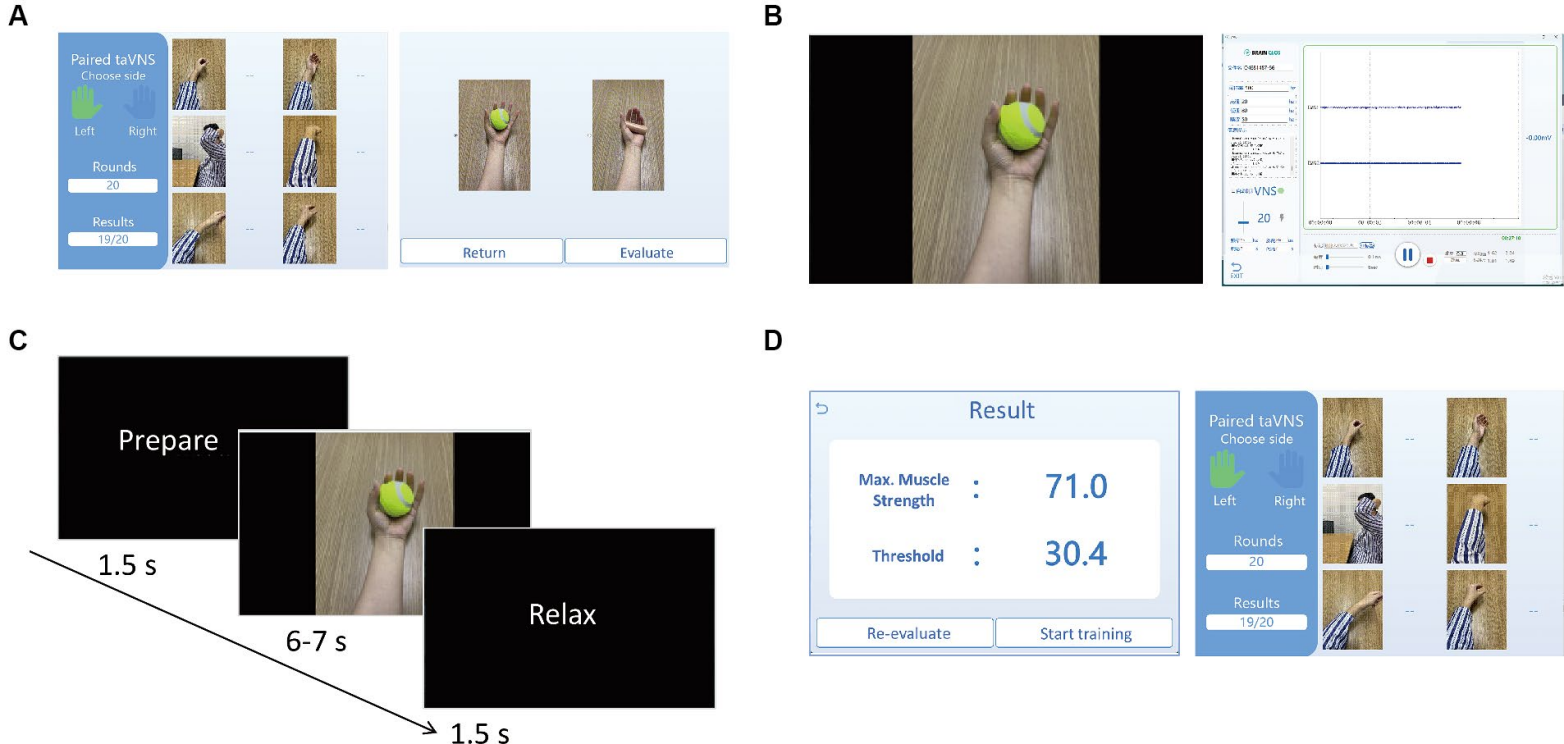
**(A)** The left interface is for selecting affected arm (left or right), the type of motor training task from six GIFs, and the number of the repeated times of the movement (i.e., rounds) by inputting the digital number under Rounds. The right interface is for selecting the sub-item (specific movement) of the motor training task. After selecting the movement and clicking Evaluate, the evaluation process will start to obtain individual threshold value of the movement for triggering taVNS. By clicking Return, it will go back to the left interface; **(B)** The left interface will be presented to the participants for the guidance of the movement training. The right interface will show the EMG waveform. The buttons on the interface can be clicked to calculate the baseline signals, determine the difficulty level, adjust the parameters of taVNS, record and save EMG data; **(C)** The timeline information of the video for guiding the movement training. For each round of the movement, it starts with 1.5 s for preparation, 6–7 s of the motor movement, and 1.5 s of rest; **(D)** After 5 times of the movement evaluation, the EMG algorithm will automatically generate the results of maximum muscle strength and the threshold for the movement training (as shown on the left interface). By clicking the Start training, the training process of the movement will start and the participant will be guided to finish 20 times of the movement according the number of Rounds. After finishing the total rounds, the right interface will show the training results, i.e., the successful rate of the movement triggering taVNS.

**Figure 3:**
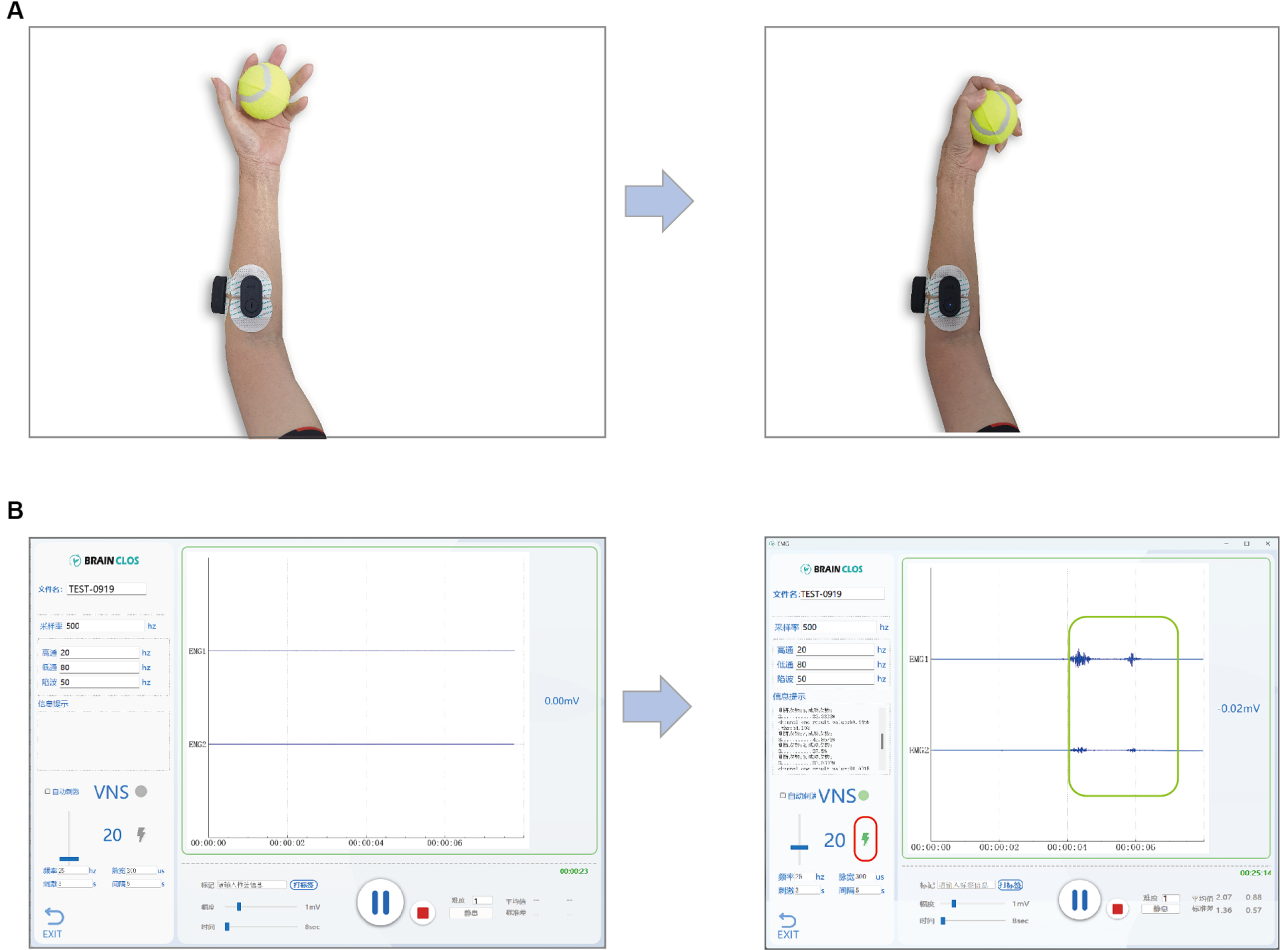
**(A)** A participant is doing motor training while recording the EMG. The left picture shows the preparation of the grip movement or the position after finish once of the movements, and the right picture shows the accomplishment of the movement. **(B)** Software show the EMG and indicate the taVNS triggered by the successful motor movement. The left picture shows the EMG while the participant is on preparation stage or rest stage. The right picture shows the EMG while the participant is doing the movement task. The green rectangle highlights the EMG signals reflecting the muscle contraction during the movement and lightening bolt symbol highlighted by the red rectangle indicates that a taVNS is triggered by the EMG feature as it passes the threshold parameters.

**Figure 4:**
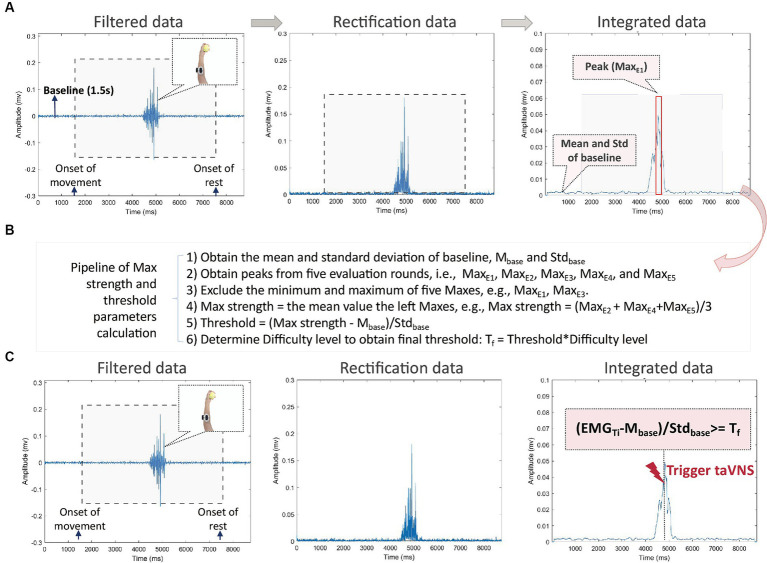
**(A)** The pipeline of data analysis during movement evaluation stage. The data of each round are marked for the onset of movement (i.e., the start of the video) and the onset of the rest (i.e., the end of the video). The baseline is defined as a 1.5-s period without any movement before the first round, such as the Baseline highlighted on the left picture. The gray rectangle marks for the period of movement (i.e., video time). Raw data are filtered (left picture), rectified (middle picture), and integrated (right picture). From the integrated data, the mean and standard deviation values are calculated from the 1.5 s of the baseline. The red rectangle highlights the peak EMG (noted as Max_E_), i.e., the maximum mean EMG value during movement; **(B)** The pipeline of maximum strength and threshold parameters calculation from evaluation data. Six steps are listed; **(C)** The pipeline of data analysis during movement training stage. The EMG (noted as EMG_Ti_) is checked online to trigger taVNS. If (Max_Ti_ – M_base_/Std_base_ ≥ T_f_, taVNS) (real or sham) will be triggered, otherwise no taVNS (real or sham) will be triggered.

As illustrated in Figure 2A, the therapist will initiate the process by selecting the specific type of movement task, specifying the desired number of training repetitions (typically ranging from 30 to 50 rounds), and choosing a sub-item of the task (Figure 2A). The evaluation process has a predetermined integer number of 5, meaning that the movement will be repeated five times. The software records, displays (as depicted in Figure 2B), processes, and decodes EMG signals for both evaluation and training phases. During the evaluation, participants will replicate the prescribed movement for five rounds, guided by the instructional video. Each round comprises preparation, movement, and a rest period (as depicted in Figure 2C). Upon completion of the evaluation, the software will process the EMG signals from the current movement task and generate an evaluation result (as shown in Figure 2D). Participants then transition to the training stage, where they repeat the same movements for the number of rounds specified earlier, as shown in Figure 2D. As demonstrated in Figures 3, 4C, successful execution of the movement by the participant, as determined by EMG features surpassing the threshold parameter, will trigger a burst sequence of taVNS. Such a round of movement will be classified as a successful one (for detailed analysis, refer to the Data Processing section and Figure 4). The software accumulates the successful movement rounds and displays the total count in the Results section (Figure 2D).

For participants in Group 1, the amplitude level of the taVNS for each burst sequence remains the same as the pre-training setting. Conversely, in Group 2 and Group 3, the amplitude level of taVNS will be set to zero. According to Dawson et al. (12), to maintain the blindness of participants regarding the experiment’s objectives, the experimenter will activate the taVNS three times using a remote control prior to each type of motor movement training.

Each session lasts approximately 45 to 60 min and the success rate of movements triggering taVNS should reach 85% or higher. The therapist will closely monitor for compensatory actions during each movement task, and participants will be instructed to rest if fatigue or compensatory actions are observed. Specific training tasks for each session will be adjusted according to the participant’s rehabilitation progress.

### Outcome measures

The primary outcome measures include assessment of treatment efficacy by evaluating motor functions via FMA-UE. It is a clinical tool to assess motor function and recovery with upper limb impairments resulting from stroke or neurological conditions in individuals. It quantifies motor skills, coordination, and reflexes through a structured examination that encompasses various subscales assessing different aspects of motor function, sensation, and coordination in the upper limbs. Drawing on the study of Dawson et al. (12), we established that a clinically significant response is defined as an upgrade of six points or more in the FMA-UE score. This basis is anchored in earlier research findings that linked an increase of 5.25 points with a substantial improvement, reflecting more than a 50% enhancement in arm functionality.

In addition to the primary outcome, this study will consider several secondary measures, including the WMFT, Brunnstrom recovery stages (BRS), Barthel Index (BI), the Hong Kong version of the Functional Test for the Hemiplegic Upper Extremity (FTHUE-HK), and EMG and electrocardiogram (ECG) features. WMFT and FTHUE-HK are designed to evaluate upper extremity motor function with neurological or musculoskeletal impairments. The BRS is a framework that describes the typical progression of motor recovery in stroke patients. It consists of six stages ranging from flaccidity to near-normal movement patterns. BI is a clinical assessment tool used to measure an individual’s ability to independently perform activities of daily living (ADLs). EMG will be recorded automatically by a computer software along with each treatment session, and the ECG features, including heart rate variability (HRV) and heart rate (HR) will be recorded before and after each taVNS treatment session as biomarkers reflecting the activation of vagal tone (33, 34).

Primary and secondary outcome measures, except for EMG and ECG, will be assessed the day before or at the latest the day after the first session and again after the 14th session. Additionally, two follow-up assessments will be scheduled, one 30 days and another 90 days following the conclusion of the last training session. To ensure consistency and reliability, each participant will be accessed by the same therapist before training, immediately after training, and during scheduled follow-up periods. EMG and ECG signals will be recorded and saved for each session.

### Equipment and parameters

The taVNS device (BC102-IV, BrainClos, Shenzhen, China), EMG (BC107, BrainClos, Shenzhen, China) and ECG (BC116, BrainClos, Shenzhen, China) used in this experiment will be provided by Shenzhen BrainClos Technology Co., Ltd. The stimulation parameters of the taVNS are as follows: a frequency of 25 Hz, a pulse width of 300us, and a stimulation intensity ranging from 0 to 6 mA. This intensity can be finely adjusted across 60 levels, with each level representing a 0.1 mA increment. The stimulation intensity will be customized based on the participant’s tolerance and comfort level. In Group 1, each EMG-triggered taVNS burst lasts for 3 s. The total pulses will depend on the number of successful movements. In Group 2, each taVNS session lasts 31.25 min, cycling through 3 s of stimulation followed by 4.5 s rest. The total number of pulses will be 18,750.

Both EMG and ECG have a sampling rate of 500 Hz, and high precision signals are obtained through a 24-bit AD converter. The two devices are designed to use Bluetooth communication with very low noise to acquire uV-level electrophysiological signals. Bluetooth minimizes signal artifacts from patient movements and avoids industrial frequency interference at 50 Hz. EMG and ECG will be preprocessed and calculated for triggering taVNS or evaluating biomarker purposes, respectively.

### EMG and ECG data processing

The EMG data will be processed in real time during the evaluation and training phases. The prepossessing steps include filtering, rectification, and integration. First, EMG data will be filtered online within the bandpass range of 20-80 Hz, along with a notch filter at 50 Hz (illustrated in the left panel of Figure 4A). Subsequently, the filtered data will be rectified (as depicted in the middle panel of Figure 4A) and integrated (as shown in the right panel of Figure 4A). During integration process, we will use a moving time window of 50 ms to calculate the mean values of the EMG signal over a 100 ms interval.

ECG realtime processing will be employed to denoise the data, identify the R-R interval, and calculate HR and HRV. HRV will be represented in both the time domain and frequency domain. The chosen index of HRV in time domain is the root mean square of successive R-R interval differences (RMSSD), which reflects the beat-to-beat variance of HR and serves as a primary measure for estimating vagally mediated changes in HRV (35). The ratio of low-frequency (LF) power to high-frequency (HF) power will be used as a HRV index in the frequency domain. This ratio may provide an estimate of the balance between sympathetic nervous system (SNS) and parasympathetic nervous system (PNS) activity.

### Threshold parameters to trigger taVNS

As shown in Figure 4B, we will promptly compute the threshold parameters based on the integrated evaluation data after the evaluation phase. The data processing involves the following specific steps:

1. Initially, we calculate the mean (M_base_) and standard deviation (Std_base_) of the integrated data collected during a 1.5-s baseline period.
2. We identify and extract the peaks from five evaluation rounds, denoted as Max_E1_, Max_E2_, Max_E3_, Max_E4_, and Max_E5_.
3. To ensure robustness, we discard both the minimum and maximum peaks among the five, for instance, eliminating Max_E1_ and Max_E3_, which correspond to the minimum and maximum peaks.
4. Subsequently, we determine the maximum strength, denoted as Max strength, by calculating the mean value of the remaining three peak values, such as Max strength = (Max_E2_ + Max_E4_ + Max_E5_)/3.
5. The original threshold is then computed using the formula Threshold = (Max strength − M_base_)/Std_base_.
6. Considering factors like performance decay and muscle fatigue during training, we derive the final threshold parameter (T_f_) as a relative threshold by adjusting the threshold with a difficulty level.

### Triggering taVNS from integrated training data

For each training movement, the EMG data will undergo the same prepossessing steps as the evaluation data. Each integrated data point (EMG_Ti_) will be processed using (EMG_Ti_ − M_base_)/Std_base_ to derive a parameter for initiating taVNS. If this parameter exceeds T_f_, taVNS will be activated.

To ensure the accuracy and reliability of the synchronization of taVNS and the motor movements (decoded as the EMG parameters), we conducted a comprehensive examination. This involved assessing the time precision of the software governing taVNS and EMG, the processing time for EMG data, the duration for triggering taVNS from the software, and the time delay of EMG signals from wireless electrodes. Importantly, none of these factors are expected to impact the synchronization of stimulation and motor movements in the current EMG-triggered closed-loop taVNS system.

### Statistical analyses

The critical level of significance for all statistical analyses will be set to *p* < 0.05. The analyses will be carried out by the Matlab and R statistical software packages.

#### Analysis of main endpoint indicators

Main indicators will be examined using analysis of variance (ANOVA), paired samples t-test, and independent samples t-test. Prior to conducting inferential statistics, Kolmogorov–Smirnov (K-S) tests will be employed to assess the normality of the measurement data. In cases where the data deviate from a normal distribution, transformation methods will be applied. Specifically, logarithmic transformation may be employed for data exhibiting extreme skewness, while the square root transformation can be adopted for data displaying moderate or small skewness. To evaluate the primary efficacy (i.e., the change in FMA-UE scores), a paired samples t-test will be performed within each group before and after the treatment sessions. This analysis will help determine if there is a significant improvement in upper limb function after taVNS treatment. The FMA-UE scores before and after the treatment sessions will be subtracted to calculate the FMA-UE improvement scores, representing the primary treatment effect. A one-way three-level ANOVA will be employed to examine the differences in the primary treatment effect among the three groups: concurrent, sequential, and control. Independent samples t-tests will be conducted to compare the primary treatment effects of the concurrent condition with the control condition and the sequential condition with the control group, thereby determining the treatment effects of the two experimental groups. Additional independent samples t-tests will be performed on the primary treatment effects of the concurrent and sequential conditions to assess the potential benefits of concurrent taVNS and movements. Wilcoxon signed-rank test and the Friedman test will be performed for the ordinal outcome measures. The effect sizes of the treatment effects in each group (Cohend’ s *d*) and those of the differences in treatment effects among three groups (Eta-squared, *η*^2^) will be documented.

#### Analysis of secondary endpoint indicators

Secondary endpoint indicators will be statistically examined by ANOVA, concurrent samples t-test, and independent samples t-test. The statistical results will be corrected based on Bonferroni’s principle as this statistical operation requires multiple comparisons, and there will be a bias of Alpha inflation. The scores for WMFT, BRS, BI, and FTHUE-HK before and after treatment sessions will be analyzed using the same approach as for the primary endpoint indicators. The muscle strength and threshold values calculated from EMG signals during the first and last sessions from the same motor movements will be subtracted and averaged for each participant. In cases where no identical motor movement between the first and last sessions, additional motor movements identical to the first session will be evaluated in the last session. ANOVA will be utilized to analyze the changes in strength and thresholds across the three groups, exploring the effects of taVNS on EMG features of motor movements. The HRV values before each session will be subtracted from those after each session and averaged for each participant. ANOVA will be employed to analyze the HRV changes across the three groups to examine the effects of taVNS on HRV. The corresponding effect sizes of secondary indicators will also be analyzed.

#### Analysis of data from follow-up evaluations

Data collected during follow-up evaluations will exclusively consist of questionnaires, with no inclusion EMG and ECG data. The evaluation of primary and secondary endpoint indicators will follow the same procedures outlined earlier during the follow-up assessments.

## Expected results and discussion

Currently, no studies have systematically examined the necessity of taVNS applied concurrently with motor training for stroke rehabilitation. Nor have studies provided direct clinical evidence for the benefits of pairing taVNS with movement by decoding EMG signals during motor training. Previous studies have indicated that pairing VNS (10–12) or taVNS (27, 28, 30) with rehabilitative training can enhance motor function recovery. Whereas a study by Wu et al. (31) demonstrated that taVNS treatment prior to regular motor training could improve motor functions. Recent research by Badran et al. (27) highlighted the benefits of movement-synchronized taVNS by decoding EMG signals compared to simple combination of taVNS and movements. However, their study was not designed with a sham control group and the number of subjects per group was limited to less than 10. In comparison, our study can validate the experimental results in a larger sample size with a sham control group. On the other hand, the results will highlight the value of the EMG-triggered closed-loop system for VNS and taVNS treatment for motor rehabilitation, which could be labor-saving compared to the original protocol by Dawson et al. (12) where a therapist constantly monitors each motor movement and trigger the taVNS manually. This study represents a pioneering effort in directly comparing the impact of concurrent taVNS with motor training to that of sequential taVNS alongside motor training.

We anticipate that upper limb motor function could be significantly enhanced in concurrent group compared to the other two groups, as indicated by the FMA-UE scores. Furthermore, we expect that the results of secondary outcome measurements will be consistent with those of FMA-UE scores. These findings are expected to provide direct evidence to support the advantages of applying taVNS concurrently with motor movement training, instead of employing them separately. On the other hand, we expect the result pattern of EMG features to be consistent with the changes in the questionnaire scores of primary and secondary measurements. After the treatment sessions, the muscle strength and the threshold of the motor movements will be increased. These results will provide physiological evidence for the rehabilitation of motor function, which will be in line with the findings from Chang et al. (28).

Regarding the features derived from ECG data before and after taVNS treatment sessions, a noteworthy reduction in HR is expected, as well as in LF/HF power ratio, and an increase in the RMSSD in the sequential group. For the concurrent group, the interleaved motor movement training may stimulate the sympathetic system, therefore we do not expect significant changes in HR, LF/HF power ratio, and RMSSD after treatment with taVNS. In the sham control group, no substantial changes are expected in ECG features. These findings, particularly in the sequential group, will validate the modulation effect of parasympathetic activity within the vagus nerve system through the transcutaneous stimulation of ABVN.

Although the ECG features can be utilized to assess vagal tone activation and the EMG features may reflect motor function progress and the sympathetic tone to some extent, these results may not sufficiently unveil the neural mechanisms underlying the rehabilitative effects of EMG triggered closed-loop taVNS on motor deficits. Therefore, additional electrophysiological assessments such as sympathetic skin responses, motor evoked potentials via transcranial magnetic stimulation (TMS), vagus somatosensory evoked potentials (VSEP) using electroencephalography (EEG) recordings (36, 37), or TMS-evoked potentials (TEP) should be considered. Designing studies to explore modifications in spinal ascending and descending tracts and circuits would provide valuable insights into the modulation of motor control and corticospinal motor networks following taVNS. Functional magnetic resonance imaging (fMRI) assessing changes in brain activity and the default mode network before and after treatment sessions or during taVNS intervention is equally crucial (38, 39). Collectively, insights gathered from these multidimensional perspectives would offer a comprehensive understanding of the mechanisms underlying closed-loop taVNS for stroke rehabilitation.

TaVNS, especially in the context of closed-loop taVNS, has the potential to integrate a broader spectrum of rehabilitation strategies beyond traditional motor physical therapy, including the application of Brain-Computer Interface (BCI) technology. Within the domain of stroke rehabilitation, BCI technology has employed motor imagery (MI) training with surface EEG recordings, incorporating visual or electrical feedback (e.g., functional electrical stimulation, FES) (40, 41). The real-time nature of the feedback makes BCI a valuable tool for promoting motor recovery. The rehabilitative benefits of BCI protocol are linked to active rehabilitation and brain neural plasticity, aligning with prior findings on the reinforcement mechanisms underlying taVNS for motor rehabilitation (9, 12, 15). Integrating taVNS as additional feedback, either alongside or following visual feedback or FES during MI and BCI training, could offer a novel intervention to enhance the overall effectiveness of stroke rehabilitation. This approach would be particularly valuable for stroke patients lacking adequate muscle strength to participate in the current study, thereby expanding the inclusivity and effectiveness of stroke rehabilitation interventions.

## Conclusion

This study will provide direct evidence for the necessity and advantages of the concurrent application of taVNS and motor movement training in the rehabilitation of upper extremity motor function after stroke. The incorporation of an EMG-triggered closed-loop taVNS system has enabled the automation and individualization of both taVNS and diverse motor training tasks—a novel approach not explored in previous research. This technological advancement holds promise for delivering more precise and tailored training interventions for patients.

## Data availability statement

The original contributions presented in the study are included in the article/supplementary material, further inquiries can be directed to the corresponding author.

## Ethics statement

The studies involving humans were approved by Medical Ethics Committee, affiliated with Zhujiang Hospital of Southern Medical University. The studies were conducted in accordance with the local legislation and institutional requirements. The participants provided their written informed consent to participate in this study.

## Author contributions

X-ZX: Writing – review & editing, Writing – original draft, Visualization, Validation, Supervision, Software, Resources, Project administration, Methodology, Investigation, Funding acquisition, Formal analysis, Data curation, Conceptualization. RL: Supervision, Project administration, Methodology, Conceptualization, Writing – review & editing, Writing – original draft, Investigation. CX: Writing – review & editing, Project administration, Methodology, Investigation, Conceptualization. SL: Writing – review & editing, Supervision, Methodology, Investigation, Data curation. MY: Writing – review & editing, Software, Methodology, Investigation. HZ: Writing – review & editing, Project administration, Methodology, Investigation, Data curation, Conceptualization. XH: Writing – review & editing, Supervision, Methodology, Investigation, Conceptualization. JM: Writing – review & editing, Supervision, Software, Methodology, Investigation. QX: Writing – review & editing, Supervision, Resources, Project administration, Methodology, Funding acquisition, Conceptualization.
